# David V procedure and hemiarch replacement in a patient with Loeys-Dietz-Syndrome and beta thalassemia minor: a case report

**DOI:** 10.1186/s13019-023-02347-6

**Published:** 2023-08-27

**Authors:** Frieda-Maria Kainz, Kathrin Freystaetter, Bruno K. Podesser, Christoph Holzinger

**Affiliations:** 1 Department of Cardiac Surgery, University Hospital of St. Poelten, St. Pölten, Austria; 2 Center for Biomedical Research and Translational Surgery, Medical University of Vienna, Vienna, Austria

**Keywords:** Loeys-Dietz-Syndrome, Hypothermia, Valve sparing root replacement, David procedure, Beta thalassemia minor, Cardiopulmonary bypass

## Abstract

We report the case of a 36-year-old European female patient presenting with a sinus valsalva aneurysm of 47 mm with moderate aortic regurgitation. Additionally, an aneurysm of the brachiocephalic trunk and multiple aneurysms of the right internal mammary artery were identified. Previous medical history included Loeys-Dietz syndrome (LDS) Type RII due to a TGF-beta receptor mutation, and beta thalassemia minor with a baseline hemoglobin of 9,3 g/dL on admission.

Reconstruction of the aortic root and hemiarch replacement was performed in circulatory arrest under moderate hypothermia. During surgery, hypothermia was required as part of the cerebral protection strategy. We aim to highlight special considerations and discuss the effects of cooling, rewarming and the use of cardiopulmonary bypass (CPB) during extensive surgery in a patient with LDS and beta thalassemia minor.

## Background

Loeys-Dietz-Syndrom (LDS) results in a generalized arterial tortuosity, a bifid uvula, hypertelorism and cleft palate. These clinical signs are caused by an overproduction of collagen, loss of elastin and the disarray of elastic fibers. These structural changes result in a weakened vascular media, leading to dilatation and dissection of blood vessels. These patients have a higher risk of aneurysms, dissection or rupture of the aorta even at young ages and smaller aortic diameters. Early recognition and treatment is of utmost importance in the management of these patients [1].

Additionally, our patient inherited beta thalassemia minor from her mother. Beta-Thalassemia is an autosomal recessive disorder with three major types primarily affecting the production of hemoglobin Beta thalassemia affects the production of beta globin chains, thus leading to impaired erythropoiesis, resulting in anemia. Beta thalassemia minor usually causes mild symptoms, but severe forms can lead to hemochromatosis, accompanied by cardiomyopathy, systolic and diastolic dysfunction of the left and right ventricle, arrhythmias and pericarditis, which is the main cause of death in older thalassemia patients. In patients with the intermedia form there is a higher risk to develop pulmonary and arterial hypertension because of the chronic hemolysis and the pro-thrombotic degenerated membrane of the erythrocytes [2].

## Case representation

**Fig. 1:**
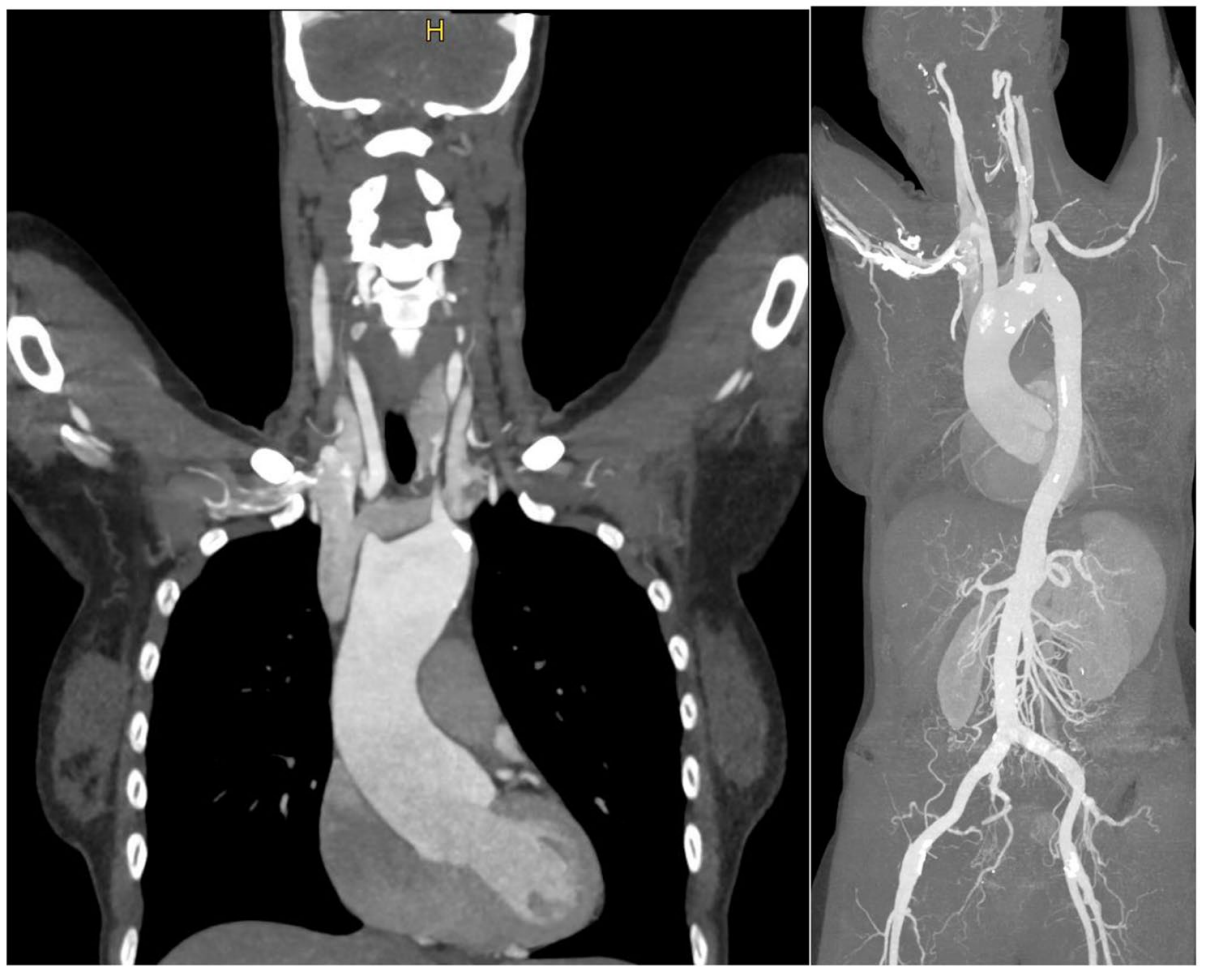
Preoperative CT scan

A 36-year-old European female patient with a sinus valsalva aneurysm of 47 mm and moderate aortic regurgitation was referred for elective surgery. The diameter of the ascending aorta was dilated to 40 mm, further aneurysms of the brachiocephalic trunk (14 × 17 mm) and multiple aneurysms of the right internal mammary artery were evident on computed tomography (CT).(Fig. 1) Previous medical history included Loeys-Dietz syndrome and beta thalassemia minor.

Hemoglobin (Hb) on admission was 9,3 g/dL, hematocrit (Hk) 30%, AT III activity 89%, transferrin saturation 13%, ferritin 11,9 ng/ml.

**Fig. 2:**
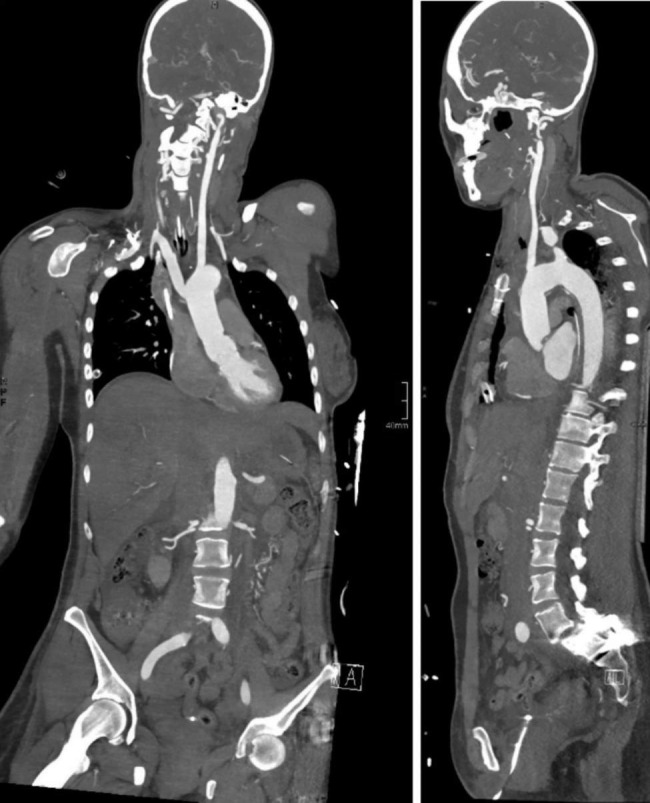
Postoperative CT scan

**Fig. 3:**
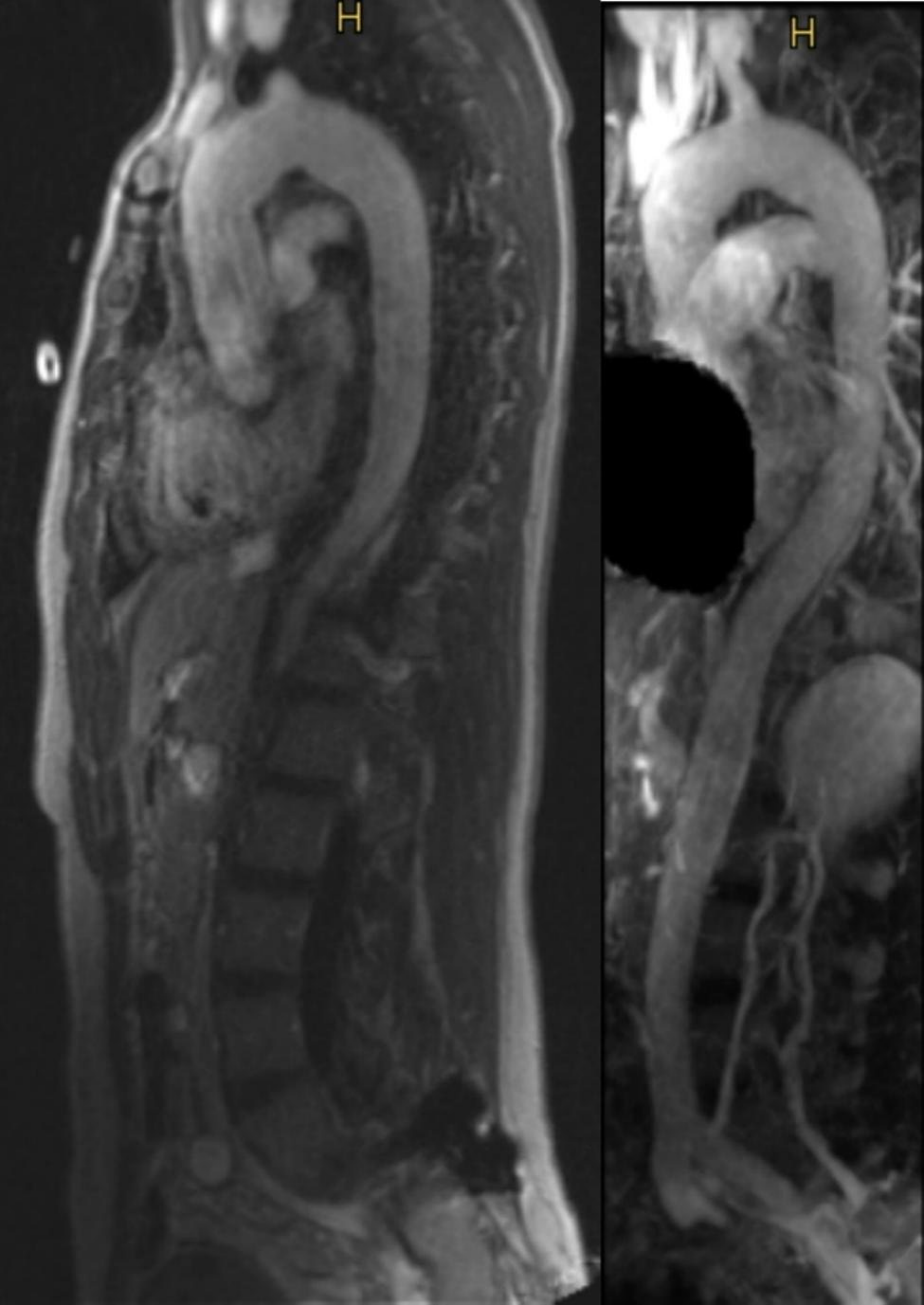
Two years: postoperative MRI control

Our patient underwent a David V operation (valve sparing aortic root replacement, VSARR) and a hemiarch replacement through a median sternotomy in moderate hypothermia and circulatory arrest. Following median sternotomy, arterial cannulation was established via the ascending aorta and venous cannulation via the right atrium, a coronary sinus catheter was inserted for retrograde cardioplegia. Cardiopulmonary bypass was commenced, the patient cooled to 29 degrees Celsius. Circulatory arrest was induced, cerebral perfusion catheters inserted into the brachiocephalic trunk and left carotid artery. In-Vivo-Optical Spectroscopy (INVOS) was used to monitor cerebral perfusion. The aortic arch was replaced with a 28 mm Dacron Hemiarch graft, the aortic root was reconstructed with a 28 mm Dacron Valsalva graft. The circulatory arrest time was 23 min, the aortic cross-clamp time 2 h and 31 min, and extracorporeal circulation time 3 h and 14 min. The aneurysms of the right internal mammary artery were excluded with multiple ligatures using 3/0 Prolene. Intraoperative transesophageal echocardiography showed a competent aortic valve with sufficient leaflet coaptation, no leaflet prolapse, and good biventricular function. The patient required ventricular pacing with temporary pacing wires due to a second-degree atrioventricular (AV) block. Postoperatively, the patient was transferred to the intensive care unit. Postoperative laboratory results showed: hemoglobin 10 g/dL, hematocrit 30,9%, AT III activity 63%. The day after surgery, AT III activity increased to 74%, fibrinogen 319 mg/dl, prothrombin time 71%, Hb 10,9 g/dL, Hk 34,0%. Extubation was delayed to the second postoperative day (POD) due to delayed emergence from anesthesia, a cerebral CT scan showed no abnormalities. Due to a third-degree AV block and continued pacemaker dependency, a permanent pacemaker was implanted on the 4th POD. She was transferred to the regular ward on the 7th POD. Echocardiography prior to discharge showed minimal central aortic regurgitation. Postoperative CT angiography of the aorta showed an excellent result (Fig. 2). The patient was discharged home on the 11th POD and remains alive and well three years after surgery. Two-year control with MRI showed an excellent long-term result. (Fig. 3)

## Discussion

Williams et al. report of 71 patients with LDS, 6 died prior to operation due to dissection or aneurysm (smaller than 4,5 cm) rupture. Aortic surgery was performed on 21 patients (14 children, 7 adults), the rest received medical treatment only. 62% of patients were diagnosed with LDS before the operation. The mean age was 16,3 +/- 12.1 years, 24% of the patients had undergone previous cardiac surgery. Remarkably, only one patient presented with aortic valve regurgitation. The most common procedure was a valve sparing aortic root replacement (VSARR). One child who underwent VSARR with aortic arch replacement required a Bentall procedure due to significant aortic regurgitation 5 months after primary surgery [1].

Aortic root dilatation can manifest at a much younger age in LDS patients and is linked to a significant risk of dissection or rupture of the aorta. For these patients, surgical options include a composite root replacement or VSARR. Both methods achieve excellent long-term results with low mortality rates [3] [4][5]. Valo et al. showed excellent survival rates, freedom from recurrent aortic valve insufficiency or the need for reoperation even in high-risk patients following VSARR. Out of 78 patients, 3% had a stroke, 1% prosthesis infection, 1% low cardiac output syndrome and 3% renal failure. After 5 years, 97% of the patients were still alive [4]. A best-evidence article from 2009 recommended the David operation for Marfan patients, acute aortic dissection and bicuspid aortic valves [5]. The advantage of VSARR is to avoid anticoagulation, which is often connected with lifestyle modifications and restrictions. Also, it increases the risk of intraoperative bleedings especially in young patients with tissue disorder with a high risk for reoperation. Tweddell et al. suggest early elective VSARR in LDS patients, when the sinus of valsalva is larger than 40 mm [3].

For patients with beta thalassemia, meticulous management of CPB is especially important to avoid hypoxia and anemia. The stress of CPB can damage erythrocytes and cause hemolysis. Exposure to negative pressure and the interface with air are the two major determinants of hemolysis in bypass circuits. To decrease the risk of morbidity and mortality, adequate transfusion of blood products, sufficient monitoring and a suitable operative strategy is key.

Adequate cerebral protection is essential for aortic surgery. Deep hypothermic circulatory arrest combined with antegrade or retrograde cerebral perfusion should be employed to minimize brain injury (evidence level B, AHA guidelines) [6]. The potential temperature range varies from 12 to 30 °C, managed by ECC, with lower temperatures linked to an increased risk of arrhythmias, coagulation disorders and bleeding. Retrograde cerebral perfusion pressure is usually 20 to 40 mmHg and antegrade cerebral perfusion pressure 50 to 80 mmHg, with a flow of 10ml/kg body weight/min. During the last 2 to 3 decades, perfusion strategies have been adapted significantly. Deep hypothermic circulatory arrest is applicable to procedures with circulatory arrest times under 40 min, achieving good neurological outcomes. By using an antegrade cerebral perfusion strategy via the brachiocephalic trunk and the left carotid artery, our patient showed no neurological deficits postoperatively. Cerebral perfusion was monitored using INVOS measurements, ranging from 25 to 30% rSO_2_ at the beginning of ECC and increasing to 50% rSO_2_ after administration of two blood units.

Most studies with antegrade arterial brain perfusion report outcomes that are comparable to or even better than those using hypothermic circulatory arrest alone or retrograde brain perfusion. Furthermore, antegrade brain perfusion reduces the duration of brain ischemia and permits the application of less profound hypothermia, which is associated with better clinical outcomes [7].

## Conclusion

To the best of our knowledge, this is the first case report highlighting the effects of cooling in a patient with beta thalassemia minor and LDS in the setting of cardiac surgery.

INVOS measurements remained stable throughout surgery and the postoperative neurological outcome was favorable, achieved with adequate blood transfusions. The antegrade cerebral perfusion via both carotid arteries and mild hypothermia at 29 °C with the head down maneuver showed a sufficient brain protection and a safe cannulation strategy.

In patients with beta thalassemia, the approach of intraoperative CPB is especially important to avoid hypoxia and anemia. The stress of the CPB on erythrocytes can cause structural damage and thus produce free hemoglobin. Exposure to negative pressure and the surface to air interface are the two major determinants of haemolysis generated by cardiotomy suction systems. To decrease morbidity, transfusion of blood products and the length of stay in ICU, a good perioperative strategy is key. As described, VSARR is feasible also in a patient with such complex comorbidities. However, special precautions should be taken, including preloading patients to a higher hematocrit, reducing blood suctioning and continuous ultrafiltration.

## Data Availability

Data sharing is not applicable to this article as no datasets were generated or analyzed during the current study.

## Abbreviations

CPB: Cardiopulmonary bypass
CT: Computed tomography
ECC: Extracorporeal circulation
INVOS: In-Vivo Optical Spectroscopy
LDS: Loeys-Dietz Syndrome
rSO_2_: Regional oxygen saturation
TGF: Transforming growth factor
VSARR: Valve sparing aortic root replacement
